# The Evolvement of OCT and OCT-A in Identifying Multiple Sclerosis Biomarkers

**DOI:** 10.3390/biomedicines11113031

**Published:** 2023-11-11

**Authors:** Vlad Constantin Donica, Anisia Iuliana Alexa, Irina Andreea Pavel, Ciprian Danielescu, Manuela Andreea Ciapă, Alexandra Lori Donica, Camelia Margareta Bogdănici

**Affiliations:** 1 Department of Ophthalmology, Faculty of Medicine, University of Medicine and Pharmacy “Grigore T. Popa”, University Street, No. 16, 700115 Iasi, Romania; vlad-constantin.donica@d.umfiasi.ro (V.C.D.); ciprian.danielescu@umfiasi.ro (C.D.); camelia.bogdanici@umfiasi.ro (C.M.B.); 2 Emergency Hospital “Dimitrie Castroian”, 735100 Husi, Romania; 3 Clinical Recovery Hospital, 700661 Iasi, Romania

**Keywords:** optical coherence tomography, optical coherence tomography angiography, OCT, OCT-A, demyelinating disease, multiple sclerosis, retinal vascular density

## Abstract

The prevalence of multiple sclerosis (MS) has been increasing among young people in developing countries over the last years. With the continuous development of new technology, the diagnosis and follow-up of these patients has received new parameters that physicians may use in their practice. This paper reviews the main biomarkers identified through Optical Coherence Tomography Angiography (OCT-A) involved in the development and progression of MS and investigates the role it may have in detecting changes to the central nervous system (CNS).

## 1. Introduction

### 1.1. Background

Multiple sclerosis (MS) is an autoimmune disease of the central nervous system (CNS) characterized by inflammation, demyelination and neuronal loss. It has a high prevalence amongst young people in developed and developing countries [1]. Recent studies found that the disease is more commonly found in the female population, a 3:1 female to male ratio, and has the highest prevalence amongst white, closely followed by black, individuals, with members of Hispanic and Asian descendance having a lower disease incidence [2,3]. Although the underlying cause is not well established, a series of genetic and environmental factors have been defined as increasing disease susceptibility.

### 1.2. Epidemiology

Visual symptoms tend to appear in ~70% of the MS population, with optic neuritis being the onset symptom in ~20% of MS cases [4]. Optic neuritis (ON) is an acute disorder of the optic nerve characterized by inflammation and demyelination. The patient suffers from vision loss, periocular pain that is triggered by eye movement, contrast and color vision deficits. Visual field defects are present, central-cecal scotomas being the most common defect. The patients may also present with relative afferent pupillary deficit that should be easily diagnosed in cases without prior episodes of optic neuritis (NON) [5]. Posterior segment examination could be normal in retrobulbar neuritis, or there could be signs of papillary edema (in papillitis).

### 1.3. Anatomy

**Figure 1:**
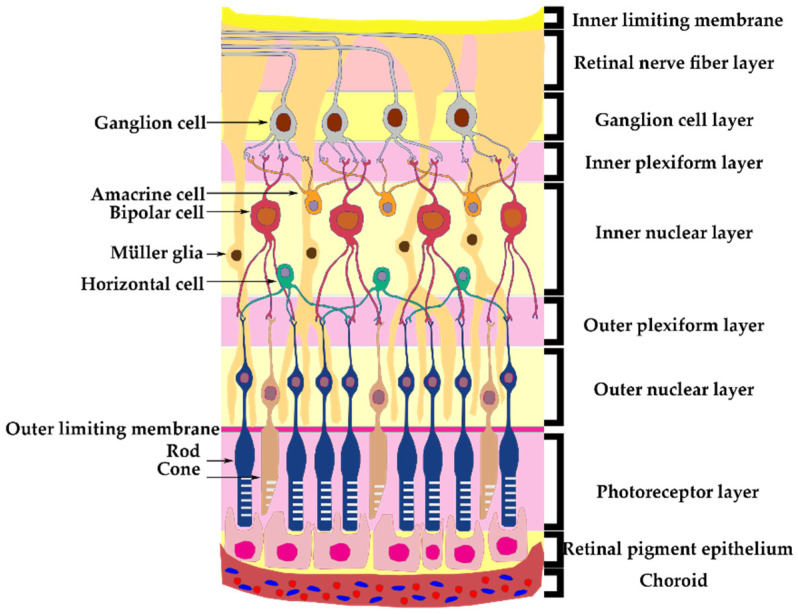
Retinal structural layers [7,8].

The retina consists of different structural layers (Figure 1). The retinal ganglion cell has the soma in the ganglion cell layer (GCL) and their axons form the retinal nerve fiber layer (RNFL), which will form the optic nerve, exit the eye and travel into the optic canal towards the optic chiasm, where the nasal part of the optic nerves will decussate and with the opposite temporal part will continue towards the lateral geniculate nuclei [6]. Lamina cribrosa (LC) is a fibrous structure that creates a barrier between the axons that form the optic nerve from the retina and the sclera in the intraocular portion.

### 1.4. Pathophysiology

The pathophysiology of MS consists of damage to the myelin and nerve fibers in the CNS. The oligodendrocytes are responsible for myelination and maintaining the saltatory function when transmitting a nerve impulse. When cells of the immune system react against the myelinated axons of the CNS, different cytokines and chemokines will be released which will infiltrate and interact with elements of the CNS causing oligodendroglial cell-mediated death, demyelination and axonal degeneration. The damage sustained by the optic pathway is a product of both acute CNS damage (ON) and trans-synaptic neurodegeneration [9]. This reaction is located posterior to the lamina cribrosa which explains the inflammatory status of the optic nerve during ON [10].

A number of studies have shown that the thickness of the RNFL and the GCL in MS eyes has the most significant difference compared to healthy eyes [11]. Even though these microstructures used to be hard to evaluate, OCT is now able to provide a thorough examination of these layers in regards to thickness and anatomical structure.

### 1.5. Optical Coherence Tomography

Optical Coherence Tomography (OCT) is a noninvasive examination of the posterior segment’s structures. It relies on low frequency interferometry to obtain high quality images of the posterior segment elements such as vitreous, retina and choroid. Due to advances in technology, these scanning methods have become higher in quality, have a faster capture rate and a deeper tissue penetration capacity.

OCT biomarkers represent imaging parameters obtained by analyzing various structures of the posterior segment such as RNFL, GCL and vascular parameters that can act as indicators of normal or pathogenic processes. They can be used to assess biological status, disease progression or to monitor treatment efficiency.

## 2. Materials and Methods

**Figure 2:**
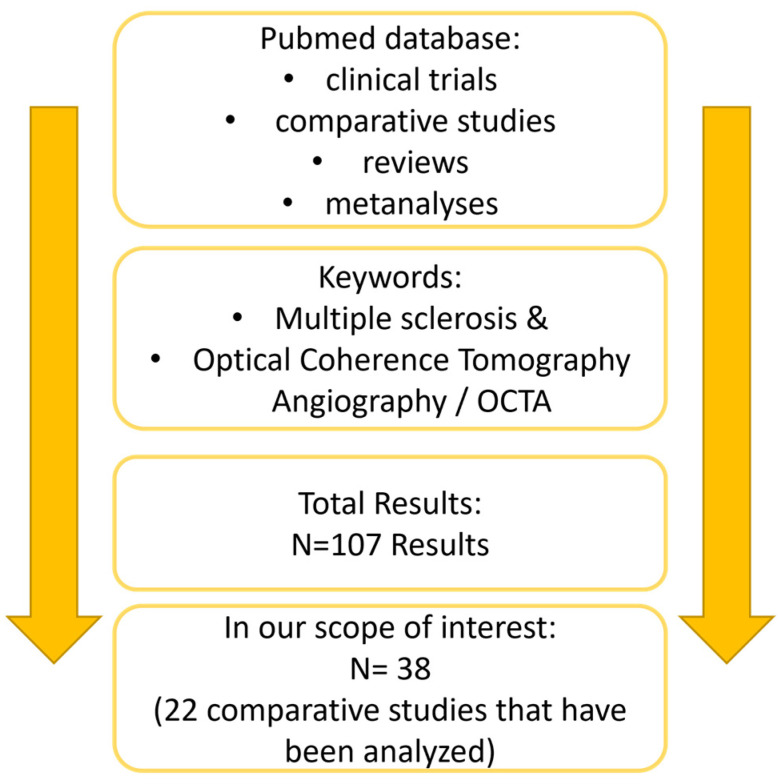
A schematic representation of the current study design.

We searched the PubMed database focusing on all published studies. We used the following keywords: OCTA; Optical Coherence Tomography Angiography; multiple sclerosis, to find relevant articles on the selected topic. We compared all appropriate articles that we found from the literature and focused on prospective and retrospective studies, metanalyses and reviews, excluding abstracts, documents, non-English articles, editorial letters, conference posters and preprints. Our search resulted in 107 results with only 40 being in our scope of interest. We found 22 comparative studies that analyze different OCT-A parameters in MS patients. A schematic representation of the study design is shown in Figure 2.

## 3. Optical Coherence Tomography Structural Parameters

### 3.1. Retinal Layers

Studies published in the last ten years have focused on thickness modifications of the peripapillary RNFL (pRNFL) and the thickness of the GCL in the macular area. Sometimes, the GCL is hard to separate from the inner plexiform layer (IPL) which is underneath, so most machines include both the GCL and the ganglion cell inner plexiform layer (GCIPL) in the report [12]. 

Saidha et al. investigated 107 patients biannually for 46 months measuring GCIPL using OCT and total brain volume using MRI. The study compared the relation between GCIPL and brain atrophy and how it differs depending on prior episodes of ON. They concluded that a thinning of 1 µm/year in eyes with prior ON results in a 0.06% rate of brain atrophy. In patients without prior ON, the thinning of 1 µm/year led to a 0.46% rate of brain atrophy. The rate of GCIPL thinning also depends on baseline values and disease relapses. They also observed that in patients with recurrent remissive multiple sclerosis (RRMS), the inner nuclear layer (INL) is thicker and microcysts were found in six patients, five of which had prior ON. Therefore, INL analysis could be used to assess the activity of inflammation in the CNS. They concluded that measuring the GCIPL using OCT could mirror the grade of brain atrophy registered by the MRI [13]. Cellerino et al. also analyzed the implications of INL thickness in MS patients establishing a correlation between younger patients, age < 51 and recent T2 lesions [14].

A large multicenter, prospective study conducted by Paul et al. followed 333 patients with RRMS over a 36-month period measuring the pRNFL and GCIPL. Their main results show that there is a faster thinning of the pRNFL in patients with <3 years disease activity compared to those with >5 years disease activity. MS patients without ON had a similar decrease in thickness as those with prior ON, suggesting that the pRNFL thinning is not necessarily a post-inflammatory consequence, but a marker of disease severity. They also suggest that there is not only axonal degeneration, but neuronal loss as well, due to GCIPL thinning. By comparing GCIPL degeneration using OCT and brain volume loss using MRI, they confirm the results of Saida et al., where the GCIPL thinning could be used to assess disease severity in the entire CNS [13,15].

The faster rate of axonal degeneration in RRMS patients in the first years of the disease was also noticed by Cilingir et al. in a study that compared 66 RRMS patients with <3 years of disease activity with a second group of 69 patients with >5 years of RRMS disease activity. They suggest that axonal degeneration is independent of inflammatory activity [16].

Cujba et al. analyzed the OCT structural parameters of RRMS, clinically isolated syndrome (CIS) and healthy eyes. They found that the neurodegenerative process affects the pRNFL and the macular structural layers in the MS ON eyes compared with CIS ON eyes. CIS ON eyes showed a significant decrease in the GCL compared with CIS NON eyes, but no difference regarding pRNFL was found between CIS and healthy patients [17].

Frau et al. conducted a study on 66 MS patients that focused on the relation between RNFL thinning, a decrease in cognitive function and brain atrophy, concluding that they are all consequences of neurodegeneration with no direct link between each other [18]. 

### 3.2. Lamina Cribrosa

There have been a number of studies that show the importance of LC measurements in patients with glaucoma. Such a feature has become more accessible because of the development of enhanced depth imaging OCT (EDI-OCT) [19]. In a study by Hamamci et al., patients with MS were divided in three groups. One group was known to have a history of modified visual evoked potentials (VEP), another had no history of modified VEP or ON and there was a third healthy control group. OCT disc analysis was performed for all patients measuring pRNFL thickness and lamina cribrosa thickness (LCT). The latter was measured using manual segmentation by two separate researchers. The results showed that LCT was lower in MS patients with a history of modified VEP compared to the healthy control group, and that pRNFL had the lowest thickness values in the same patient category. They concluded that the results were statistically significant regarding the two parameters; however, larger longitudinal studies should be performed in order to verify these results [20].

### 3.3. Vascular Changes

The vascular changes that appear in MS have been analyzed in different types of studies. Retinal perivenular abnormalities that appear in regions without oligodendrocytes in ON patients have been found to have a high incidence in developing MS. These vascular modifications appear to be a separate event, and suggest that the inflammatory episodes can occur independently from demyelination [21]. 

Moreover, different studies suggest that endothelin-1, a vasoconstrictor peptide produced intrathecally, could be responsible for the vascular abnormalities [22]. Endothelin-1 levels were measured in MS patients and results showed that the internal jugular vein blood had significantly higher levels in MS patients compared to a healthy control group. This implies the role that the vasoconstrictor peptide has in cerebral hypoperfusion which appears in MS patients [23]. Furthermore, MS patients with high levels of endothelin-1 receptor in their cerebrospinal fluid (CSF) had a lower visual recovery from ON compared with those without [24]. This comes to support some authors claims that MS is a CNS disease with a strong vascular component [25].

### 3.4. Choroidal Thickness

The vascular changes that affect MS patients will lead to modifications of the choroidal microarchitecture. By using EDI-OCT, measurement of the choroidal layer has become more accessible in recent years, with choroidal thickness (CT) becoming a new biomarker involved in the follow-up of MS patients [26]. Esen et al. measured the CT in 68 MS patients and compared it to healthy controls. They concluded that all MS patients present modifications of the choroidal architecture, with ON eyes having a thicker CT, whilst NON eyes have a thinner CT compared to healthy controls [27]. Masala et al. performed a similar study in which the results were not statistically significant when comparing CT in MS and healthy eyes. However, all patients with a history of ON presented a thicker CT [28]. In a study by Garcia-Martin et al., an automated method of measuring CT in MS patients had been used. The advantages of this method were the large area of analysis, other manual studies having only certain points of measurement, the automatic determination removing the human source of error and increasing the accuracy and reproducibility rates. They concluded that MS eyes have a thinner CT compared with healthy eyes, the values being of a higher difference as the measurements approach the peripapillary area [29].

## 4. Optical Coherence Tomography Angiography Vascular Density Parameters

Optical Coherence Tomography Angiography (OCT-A) is a method of visualizing in vivo the vascular architecture of the superficial and deep plexus of the retina and the choriocapillaris plexus. By realizing several sequential OCT scans, the method uses erythrocyte blood flow as a method of mapping the retinal vessels. It is a non-invasive technique that has become a subject of intense research in recent years [30,31]. 

The parameters gained from this investigation can quantify the vascular density (VD) in different areas such as the area surrounding the optic disc, the macular area and at different levels such as the retinal superficial vascular plexus (SVP), deep vascular plexus (DVP) and the choriocapillaris plexus. Studies using different machines were used to compare these values in healthy and MS eyes with or without prior ON. The SVP contains blood vessels that form the inner retina (from the RNFL to the INL), while the DVP is formed from vessels found in the outer retina. Many studies have recently tried to highlight the differences in OCT-A parameters between healthy and MS patients (Table 1).

Farci et al. analyzed the capillary flow density (CFD) at different vascular levels in MS patients, both with a history of ON and without, and compared them to a healthy control group. The results showed an increase in the CFD in MS patients at all levels except for the SVP, regardless of their ON history. Therefore, vascular modifications that occur at these levels appear to be independent of the inflammatory status [32]. 

When correlating with retinal structural layers, the atrophy of the RNFL and GCIPL may lead to a loss of vascularization at this level which could explain the lower vascular density in MS patients in the SVP [32].

Montorio et al. conducted a study using a widefield imaging device which allowed measuring CFD, in MS patients, in the peripheral retina where the ganglion cell complex is normally reduced and showed that there is a decrease in VD even in the periphery. This suggests that vascular atrophy in the SVP may occur independently from GCIPL atrophy [33].

**Table 1:** Published studies that analyze OCT-A values between MS eyes and healthy eyes.

Author and Year	Participants and Method	Analyzed Structures	Sub Groups	Study Limitations	Key Findings
Gao et al. [34] 2023	72 MS eyes vs. 74 healthy eyes	ORT CC VD CT CVI	MS ON vs. MS NON	Not longitudinal	MS NON patients had decreased CC VD and CVI. No difference in ORT and CT between them. In MS patients, no correlation between OCT-A parameters and EDSS score were found.
Montorio et al. [33] 2022	33 MS eyes vs. 35 healthy eyes	FAZ area SVP VD DVP VD	-	Small lot size No subgroups Not longitudinal	Decreased SVP VD in periphery where RNFL and GCIPL are normally decreased. FAZ area increased in MS patients. No significant DVP VD differences
Khader et al. [35] 2021	10 MS ON eyes vs. 10 MS NON eyes vs. 10 healthy eyes	RNFL GCC ONH VD	MS ON vs. MS NON	Small lot size Not longitudinal	Decrease of VD around ONH, in SVP, DVP, more significant in ON patients. Lower pRNFL and GCC thickness corelates with decreased VD surrounding the ONH.
Balikci et al. [36] 2021	164 MS eyes vs. 114 healthy eyes	FAZ area SVP VD DVP VD RNFL GCL	MS ON vs. MS NON	Not longitudinal	No significant difference regarding VD. No significant difference for structural parameters for MS groups. Correlation between structural OCT parameters and OCT-A vascular modification.
Jesus et al. [37] 2021	45 MS eyes vs. 45 healthy eyes	Choroidal VD CC VD	MS ON vs. MS NON	Not longitudinal No correlation with other parameters Manual method	Decrease in VD in both Choroid and Choriocapillaris layers 500 to 1500 µm from the fovea.
Murphy et al. [38] 2020	Cross-sectional 43 MS ON patients with 92 visits vs. 14 MS NON patients with 24 visits	IED between: RNFL GCC VD in SVP	MS ON >3 m. vs. MS ON 3–12 m vs. MS ON 1–3 y vs. MS ON >3 year vs. MS NON	-	Significant IED difference in VD of SVP in MS ON vs. MS NON. Loss of VD in SVP is better correlated with visual function than GCIPL loss. IED post ON in GCIPL appear up to 3 months after the ON episode. IED post ON in VD in SVP appear at 1 year after the ON episode.
Cordon et al. [39] 2020	92 MS eyes vs. 149 healthy eyes	VD in SVP	MS ON vs. MS NON MS < 5 years vs. MS > 5 years	Not longitudinal	Decreased SVP in MS patients especially in patients with disease activity > 5 years.
Jiang et al. [40] 2020	123 MSNON eyes vs. 36 MSON eyes vs. 198 healthy eyes	VVD SVP VD in SVP, VVD DVP VD in DVP whole retina plexus. Retinal tissue volume. RNFL GCL INL OPL	MSON vs. MSNON	The VD is calculated after removing the large vessels from the analysis, making the VD values unable to be compared with others from different studies of the same type Not longitudinal	VVD is significantly increased in MS NON compared to healthy patients in the deep and whole retinal vascular plexus. VVD in MS ON is significantly increased compared to MS NON and healthy patients. VD from the SVP and whole retina plexus in MS NON are increased compared to healthy eyes. Retinal tissue volume, RNFL and GCL are decreased in MSON and MSNON compared to healthy eyes. INL and OPL thickness is decreased in MS ON compared to healthy eyes. VVD has a better correlation with visual function and disease duration than VD. The correlation between VVD and structural parameters offer the capacity to distinguish between MS-ON, MS-NON and control groups with a higher specificity than without the vascular parameters.
Cennamo et al. [41] 2020	40 IDE eye vs. 20 MS eyes vs. 30 healthy eyes	RNFL GCL VF SVP VD DVP VD CCP VD ONH VD	IED vs. MS	Not longitudinal Small lot size	No correlation between OCT-A parameters and neurological symptoms or visual field modifications. GCL modifications correlate with VD in the SVP and around the ONH. RNFL modifications correlate with VD surrounding the ONH. VD in patients with an IDE was significantly lower when compared with MS or healthy eyes. VD in the SVP and surrounding the ONH was lower in MS eyes compared to the healthy ones. VD surrounding the ONH was lower in MS eyes compared with those with IDE. DVP and CC showed no significant modifications between the groups.
Farci et al. [32] 2020	94 MS eyes vs. 37 healthy eyes	SVP VD DVP VD CC VD ONH VD GCIPL RNFL	MS ON vs. MS NON	VD around ONH only in MS, not in healthy eyes Not longitudinal	Increased VD in DVP, CC in MS patients regardless of ON history. Decreased VD in SVP in MS ON vs. MS NON. No correlation between GCIPL and VD.
Ulusoy et al. [42] 2020	40 MS eyes vs. 48 healthy eyes	RNFL SVP VD DVP VD ONH VD	MSON vs. MSNON	Not longitudinal Small lot size	SVP VD was lower in MS vs. healthy eyes especially in ON eyes. DVP VD showed no significant differences between groups. ONH VD was significantly lower in the inferior and temporal quadrants in MS compared with healthy eyes, especially in MSON. In SVP and DVP, there was no significant difference between ON and NON eyes. Positive correlation between OCT-A parameters and disease length.
Yilmaz et al. [43] 2020	94 MS eyes vs. 122 healthy eyes	FAZ area SVP VD DVP VD MT FT ONH VD	MSON vs. MSNON	Not longitudinal	No differences in FAZ area between groups. Inverse correlation between VD and FAZ. Positive correlation between SVP, RNFL and MT. Positive correlation between DVP and FT
Murphy et al. [44] 2019	Cross-sectional 201 MS eyes vs. 97 healthy eyes	RNFL GCL SVP VD DVP VD	MS ON vs. MS NON	Advanced disease patients were not included Not longitudinal	VD is decreased in MS patients regardless of ON status. In MS patients, DVP decrease was correlated with ONL thickness. SVP VD decrease was higher in MSON vs. MSNON. SVP decrease was correlated with RNFL and GCIPL. SVP decrease was more significant in patients with longer disease activity.
Feucht et al. [45] 2018	83 MS/CIS eyes vs. 100 healthy eyes	SVP VD DVP VD CC VD pRNFL GCL macular volume INL + OPL thickness	MS vs. CIS ON vs. NON	Not longitudinal	VD in SVP and DVP decreased in ON, ON, NON and healthy eyes showing comparable values. pRNFL, GCL, MT macular thickness were lower in ON vs. NON. No IED regarding OCT-A parameters. In MS eyes in both ON and NON, OCT-A values are correlated with OCT structural parameters. ON occurrence does not correlate with CC VD modifications. High values of CC VD in MS patients were correlated with previous relapse occurrence.
Spain et al. [46] 2017	68 MS eyes vs. 55 healthy eyes	ONHFI RNFL GCL	MSON vs. MSNON	Not longitudinal	No correlation between ONHFI and OCT structural parameters. When combining ONHFI and OCT structural parameters, nerve damage is diagnosed more accurately.
Lanzillo et al. [47] 2017	100 MS eyes vs. 92 healthy eyes	Macular VD RNFL GCL	MSON vs. MSNON	Not longitudinal	The correlation between VD and OCT parameters was statistically significant except for the foveal area. VD from MS eyes was significantly lower compared to healthy eyes. Inverse correlation between OCT parameters and disease severity. No correlation was found between VF and OCT angiography values.
Bhaduri et al. [48] 2016	105 MS eyes vs. 24 healthy eyes	RNFL ONH VD	MSON vs. MSNON	Not longitudinal Patients were analyzed by disease severity, not disease activity.	Decrease in VD surrounding the ONH is significantly correlated with disease severity. Propose that the central vascular modifications are secondary to peripheral phlebitis-related modifications.
Wang et al. [49] 2014	52 MS eyes vs. 21 healthy eyes	ONHFI perifoveolar FI	MSON vs. MSNON	Small lot size Not longitudinal	ONHFI is decreased in MS eyes compared to healthy control group, especially MSON eyes. Perifoveolar FI shows no significant differences between the analyzed groups

CC: choriocapillaris; CIS: clinically isolated syndrome; CT: choroidal thickness; CVI: choroidal vascular index; DVP: deep vascular plexus; EDSS: expanded disability status scale; FAZ: foveal avascular zone; FI: flow index; FT: foveal thickness; GCC: ganglion cell complex; GCIPL: ganglion cell–inner plexiform layer complex; GCL: ganglion cell layer; IDE: isolated demyelinating episode; IED: inter-eye differences; INL: inner nuclear layer; MS: Multiple Sclerosis; MT: macular thickness; NON: no prior optic neuritis; OCT-A: Optical Coherence Tomography Angiography; ON: prior optic neuritis; ONH: optic nerve head; OPL: outer plexiform layer; ORT: outer retina; pRNFL: peripapillary retinal nerve fiber layer; RNFL: retinal nerve fiber layer; SVP: superficial vascular plexus; VD: vascular density; VF: visual field; VVD: volumetric vascular density.

### 4.1. Optic Nerve Head Perfusion

Studies regarding optic nerve head (ONH) perfusion using OCT-A have been around since 2014. Wang et al. analyzed the ONH flow index (ONHFI) using a prototype scanning method and compared MS eyes with healthy ones. The results showed a significant decrease in ONHFI amongst the MS population especially in eyes with prior ON episodes. They also analyzed the parafoveal flow index with no significant differences between the groups [49].

ONHFI was also researched in a study by Spain et al. where they compared the ONHFI of MS eyes with healthy eyes. The results showed a decreased ONHFI in MS patients, especially when there was ON history. However, the results showed no correlations with structural OCT parameters or visual function. They concluded that combining ONHFI with OCT structural parameters provided a better accuracy in diagnosing nerve damage [46].

Smaller studies analyzed the perfusion surrounding the optic nerve head (ONH) and the findings suggested that there is a significant VD reduction in the SVP and DVP in MS eyes compared to healthy eyes, regardless of their ON history, which is correlated with a lower pRNFL thickness [35]. 

In glaucoma, the decrease in ONHFI has been proven to correlate with visual field defects [50], while in MS this correlation has not been found. Cennamo et al. analyzed the VD of the macular SVP, DVP, CC and the area surrounding the ONH of MS patients, patients with an isolated demyelinating episode (IDE) and healthy patients. They found no correlation between these parameters and neurological symptoms or visual field modifications. Regarding structural OCT values, they found that the GCL modifications correlate with VD in the SVP and around the ONH while RNFL modifications correlate with VD surrounding the ONH. The VD in patients with an isolated demyelinating episode was significantly lower when compared with MS eyes or healthy ones, while the VD in the SVP and surrounding the ONH was lower in MS eyes compared to healthy ones. The VD surrounding the ONH was lower in MS eyes compared with those with IDE. DVP and CC VD showed no significant differences between the investigated groups [41].

Ulusoy et al. measured the peripapillary VD noting that there is also a significant decrease in the inferior and temporal quadrants when compared to healthy eyes. While they found no significant VD differences in the DVP between ON and NON eyes, they did find a correlation between parameters and disease activity [42].

Bhaduri et al. studied the VD surrounding the ONH and compared it to disease activity and pRNFL in both MS patients and controls. Their study showed a decrease in VD regardless of the ON status of the patients which only correlated with disease severity. They propose that central changes in the vascular architecture are secondary to peripheral phlebitis-related modifications but were unable to correlate these findings due to limitations in OCT-A protocols [48].

### 4.2. Macular Area Perfusion

The macular area has been a major area of analysis. Lanzillo et al. compared the VD in the macula region between healthy and MS eyes and noted that there is a significant correlation between VD and OCT parameters except for the foveal area and that the VD from MS eyes was significantly lower than the VD from healthy eyes [47].

### 4.3. Superficial Vascular Plexus Vascular Density

In a study by Murphy et al., the VD from the SVP and DVP was compared between MS patients and healthy control groups, observing that there is a decrease in SVP VD in MS patients. These values appear to be even lower in MS patients with ON compared to those without. There were no significant changes found in the DVP; however, a correlation between DVP and INL thickness had been reported. While associations between lower SVP VD and disease duration and severity could be drawn, this was not the case for DVP [44].

### 4.4. Deep Vascular Plexus Vessel Density

Feucht et al. compared MS patients, CIP and healthy patients and found that there are decreased values in VD in the SVP and DVP in ON eyes whilst NON and control eyes show comparable values. No differences regarding the VD of the CC were found; however, increased CC VD was correlated with recent disease activity. OCT structural parameters were lower in the ON group compared with NON eyes and they correlated with OCT-A VD values. No IED regarding OCT-A parameters was found [45]. 

### 4.5. Volumetric Vascular Density

In a cross-sectional study, Jiang et al. defined volumetric vascular density (VVD) as a new parameter obtained by reporting the vessel density of the plexus to the corresponding tissue volume. The vessel density is calculated after removing the large vessels from the analysis, making the vessel density values unable to be compared with others from different studies of the same type. VVD is significantly increased in MS NON vs. healthy patients in the deep and whole retinal vascular plexus. VVD in MS ON is significantly increased compared to MS NON and healthy patients. Unlike other reports, vessel density from the superficial and whole retina plexus in MS NON are increased compared to healthy control groups. Retinal tissue volume, RNFL and GCL are decreased in MSON and MSNON compared to healthy patients. Combined INL and OPL thickness was decreased in MS ON compared to control groups. They suggest that VVD has a better correlation with visual function and disease duration compared to VD. Furthermore, the correlation between VVD and structural parameters offers the capacity to distinguish between MS-ON, MS-NON and control groups with a higher specificity than without the vascular parameters [40].

### 4.6. Choroidal and Choriocapillaris Vascular Density

Using OCT-A, Jesus et al. measured the VD in the choroid and choriocapillaris layers. They compared 45 eyes of 45 MS patients with a 45 age-matched control group, analyzing the VD surrounding the fovea up to 1500 µm. Their study showed a statistically significant decrease in VD in both layers in the foveal and parafoveal regions, from 500 to 1500 µm. Moreover, patients with a history of ON showed an even greater VD decrease, especially in the choroidal layer [37]. These findings, although not correlated with other parameters except for axial length, incline to support the hypothesis of a choroidal ischemia that leads to retinal degeneration in MS patients.

Gao et al. measured the CC VD and choroidal VD and compared between MS and healthy eyes, finding that MS NON patients presented decreased values, highlighting the role the outer retina (ORT) plays during disease activity. ORT thickness and CT had no differences between the MS and control group, and no correlation could be made regarding OCT-A parameters and disease activity [34].

## 5. Inter-Eye Differences

Murphy et al. performed a cross sectional study to compare inter-eye differences (IED) in RNFL, GCL, average macular thickness (AMT) and superficial vascular plexus density between MS patients with and without a history of ON. They concluded there is a larger IED in all parameters for MS patients who suffered an ON episode compared to those who did not. The superficial vascular plexus showed larger IED values for patients with >1 year since the ON episode, whilst those <1 showed lower differences [38]. This could suggest that the vascular changes that affect the retinal superficial plexus are not directly related to the inflammatory episode. Cordon et al. confirm these results with their study in which the SVP in MS patients presented a lower density than in healthy control groups especially in cases with disease activity >5 years. Therefore, this reaffirms the fact that the modifications in the SVP architecture are unlikely to be caused by the episode of ON, but by disease activity itself [39].

## 6. Foveal Avascular Zone

The foveal avascular zone (FAZ) is an area with no vessels that is vital for normal visual function. Vascular modifications that take part in this area have been shown to have a significant purpose in decreasing normal visual function. FAZ enlargement can be measured using OCT-A, and has been studied in both the SVP and DVP to better understand the modifications on the macula at these levels [51]. Yilmaz et al. found no significant differences between MS and healthy patients regarding the FAZ area, perimeter or circulation. However, they noticed an inverse correlation between VD and the FAZ area at all levels [43]. These findings are also supported by Balikci et al., who also found no significant differences between healthy and MS eyes regarding FAZ parameters [36]. On the other hand, Montorio et al. found larger FAZ areas in MS patients which contradicts the previous study. However, a reason for their findings may lie in the fact that they included a small lot size [33].

## 7. Differential Diagnosis

In spite of all MS studies, there are areas in which other diseases have a similar symptomatology and the signs mirror MS. In these cases, OCT should be able to help differentiate between such instances and guide physicians until lab results with positive antibodies are confirmed [52].

### 7.1. Multiple Sclerosis and Neuromyelitis Optica Spectrum Disorder

Neuromyelitis Optica Spectrum Disorder (NMOSD) is a demyelinating disease of the CNS, that targets aquaporin 4 (AQP4), an astrocyte water channel protein. The expression of these receptors is highest in the CNS, but it can also be found in other organs. The lesion is formed when an AQP4-IgG enters the CNS via the blood–brain barrier, binding to the AQP4 receptor, triggering the complement against the astrocytes and causing cell death [53]. In the retina, the AQP-4 are found in Müller cells, which are located in the INL layer [54]. These support the findings by Fu et al., where the INL of ON NMOSD eyes was significantly thicker compared to healthy eyes [55]. OCT-A parameters have even been able to provide diagnosis capability in order to differentiate between different demyelinating diseases (Table 2).

Liu et al. measured RNFL and GCIPL thickness, macular VD and perfusion density in MS patients, Neuromyelitis Optica Spectrum Disorder (NMOSD) patients and healthy patients and compared between them. They found that OCT structural parameters are the lowest in NMOSD patients, especially in those with a history of ON. No significant differences were found between the MS NON and NMOSD NON group when analyzing RNFL and GCIPL. The most capable parameters that could discern between the two diseases were the nasal outer quadrant and the inferior outer quadrant [59]. Lee et al. confirmed the previous findings regarding NMOSD patients that had a larger decrease in RNFL, GCIPL and SVP VD than the MS group. Moreover, they compared the VD surrounding the ONH between the three groups, observing that it was significantly decreased in the NMOSD group. They also found a strong correlation between the decrease in visual function and the ONH VD [56]. 

Rogaczewska et al. measured the radial peripapillary capillary (RPC) density and compared it between MS and NMO eyes and concluded that there is a positive correlation between radial peripapillary VD and RNFL thickness in MSON, MSNON and NON NMOSD groups. The RPC in temporal, nasal and superior-temporal was decreased in MSON compared with MSNON eyes [60]. Comparing ON and NON eyes, the reduction of RPC VD in MS showed temporal and nasal modifications, whereas in NMOSD, seven out of eight parts were significantly affected [58].

Regarding the FAZ area, Aly et al. found that ON patients from both diseases had a decreased VD and an increased FAZ area, while NON NMOSD also presented higher FAZ parameters compared with NONMS and healthy eyes. These findings were even correlated with worse disease scores. Serum neurofilament light chain (sNfL) and serum glial fibrillary acidic protein (sGFAP) are new biomarkers that have been shown to have increased values in demyelinating diseases. Increased sNfL values were correlated with worse disease activity in NMOSD and MS whilst increased sFGAP levels were correlated with worse disease activity only in NMOSD and not in MS. However, they found no correlation between them and OCT-A or OCT parameters [57].

### 7.2. Multiple Sclerosis and Myelin Oligodendrocyte Glycoprotein Antibody-Associated Disease

Myelin oligodendrocyte glycoprotein antibody-associated disease (MOGAD) represents the newest addition to the inflammatory demyelinating diseases of the CNS, being only recently defined [61]. OCT has been reported to differentiate between an MS or MOGAD cause for an undiagnosed ON. Chen et al. found that MOGAD patients with ON have a significantly higher increase in thickness of the pRNFL compared to the MS ON patients [62]. In a study by Roca et al., MOGAD patients with a normal VA and ON history presented worse OCT parameters than NMOSD patients with normal VA and ON history. However, VA prognosis is worse in NMOSD than in MOGAD and the previous finding may be related to the inflammatory mechanism involved [63]. Yu et al. found that MOGAD ON patients presented decreased pRNFL, GCIPL and macular and peripapillary VD which correlate with a worse visual function, whilst they found no modified OCT structural or vascular parameters between healthy and MOGAD NON patients [64].

## 8. Visual Evoked Potentials

Visual Evoked Potentials (VEP) have long been used to assess the damage that the visual pathway underwent during ON in MS. VEP and OCT parameters are complementary and can be used to assess MS activity [10,65].

While OCT may help assess between different MS subtypes based on the degree of axonal loss (pRNFL thickness) and neurodegeneration (GCL thickness), VEP is able to provide information regarding visual recovery and prognosis. OCT and VEP can also help determine the location of the inflammatory response (OCT in anterior visual path; VEP in posterior visual path). Behbehani et al. proposed that for unilateral ON, pRNFL and GCIPL have a superior sensitivity, whilst for bilateral ON, 5th percentile VEP are more appropriate [66]. In another study, they concluded that VEP had a higher prevalence (56%) in detecting visual pathway lesions compared with OCT (48%) in patients with early RRMS [67].

Chilin’ska et al. found a significant correlation in MS patients between pRNFL, a history of ON and prolonged p100 wave. Whereas the wave amplitude may recover in time suggesting that the demyelination ended, the p100 elongation has a permanent status because of the neurodegeneration [68]. 

Ava et al. found a negative correlation between VEP and vascular OCT-A parameters such as ONH VD and RPC VD. They concluded that the involvement of the VD surrounding the ON in the inflammatory episode is expressed by a decrease in VD which correlates with prolonged p100 waves [69].

Multifocal VEP (mfVEP) are a separate type of investigation having separate responses divided into numerous sectors of the visual field [70]. In MS ON eyes, a stronger correlation was noticed between mfVEP amplitude and GCIPL thickness than between mfVEP amplitude and RNFL thickness. The edema and other inflammatory modifications of the RNFL have been proven to last longer, while GCIPL are less known to be affected by them [71]. Using mfVEP to measure recovery after an ON episode, the findings showed that the fastest time was 3 months after the initial episode, and that the amplitude is an early marker in assessing permanent axonal damage [72].

Despite the advantages the mfVEP offers, the test is more time-consuming and technically demanding; therefore, it has not replaced the standard VEP.

## 9. OCT and Treatment Efficiency

With the continuous development of new immunomodulatory drugs, the need for identifying new parameters to measure drug efficiency increases. Kal et al, measured choroidal thickness using EDI-OCT and showed that patients under Fingolimod have a normal CT in the sub foveal area, while it is decreased beyond 1000 µm to the nasal and 1500 µm to the nasal and temporal. They also compared newly diagnosed MS patients and found CT to be decreased in all quadrants compared to healthy eyes [73]. Karaküçük et al. compared the VD between healthy patients, MS patients with less than 6 months of Fingolimod and MS patients with more than 6 months under Fingolimod. They found that the FAZ area is significantly increased in patients with more than 6 months of treatment. While the SVP VD did not provide any significant differences between the groups, the DVP VD was significantly higher in MS patients with <6 months of treatment. The central macular thickness was lower in the group with >6 months of treatment than the other groups [74].

## 10. Discussion

The pathological modifications of the CNS have been studied in MS patients in order to gain insight into disease activity, progression and visual outcomes. The advantages of in vivo, non-invasive imaging that OCT and OCT-A are able to offer in the study of the anterior visual pathway has given rise to a plethora of recent and ongoing studies that are searching for new biomarkers and existing correlations between them, in order to establish new, individual types of monitoring for MS patients [75,76]. The main differences between our paper and previous reviews are our approach, highlighting the way structural and vascular parameters have evolved through OCT and OCT-A, the importance they have in differentiating between different demyelinating CNS diseases and their progression and how they contribute in the understanding of MS activity in the anterior optic path.

The 2017 revision of the McDonald criteria for an MS diagnosis proposes that further research should be focused on the involvement of the optic nerve, validation in diverse populations and incorporation of advanced imaging, neurophysiological and body fluid markers. OCT structural parameters and OCT-A vascular values fit into two of these categories, while offering a multitude of biomarkers that are still yet to be fully explored [77].

This review focused on both older studies regarding layer thickness and consistency as well as new vascular modifications such as retinal vascular plexus density or optic nerve head perfusion. Despite the fact that most studies were conducted on different OCT devices with differences in image capture, area of analysis, segmentation and report generation, the general agreement was that there is a significant decrease in pRNFL and GCIPL layers in MS patients, with a higher occurrence in ON eyes compared to NON eyes. In-depth analysis of the INL has been able to correlate with disease activity and the number of ON episodes, that has become a useful tool in assessing the prognosis and clinical disability status. Regarding the vascular parameters, the majority of studies reported a decrease in SVP density in MS patients, which was found both in ON and NON eyes which is probably explained by the decrease in layer thickness at these levels. Lower ONH perfusion rates were reported as well and further studies may provide insight into the pathophysiology of ON regarding both the inflammatory episode and the possible systemic vascular manifestations. While some studies support FAZ enlargement in ON patients [33], others have found no differences between MS and healthy eyes regardless of their ON status [43,59].

In the search for new imagistic biomarkers that could become key factors in monitoring MS activity, we believe that investigative reports which focus on values obtained by dividing the OCT-A vascular and OCT structural parameters between each other could offer a better perspective of every patient’s distinctive inflammatory profile, by comparing them over time and to normative databases. The combined analysis of such values has given rise to new entities such as VVD, which offers a more comprehensive assessment of the patient’s individual disease activity having correlated OCT-A values with disease progression, clinical status and general prognosis [40,78]. This may explain why other studies have had a lower correlation between OCT parameters and clinical status or disease activity. New future directions will target areas such as ultra-widefield OCT in order to study the vascular activity in the periphery of the retina and gain previously unobtainable parameters regarding disease progression [79].

The use of multiple devices based on both Swept-Source OCT and Spectral Domain OCT may act as a control agent for the segmentation and report-generating capacity of different software analysis. Iftikhar et al. found that artefacts are common among OCT-A imaging and can have an impact on VD parameters [80].

OCT-A has shown its capacity to distinguish between different CNS demyelinating diseases such as MS, NMOSD and MOGAD [56,57,58,59,60,61,62,63,64]. Larger acute-stage ON patient cohort studies are required to test if OCT-A has the sensitivity to differentiate between diseases and establish the stage of activity [81].

A limitation found in most of these articles is the lack of longitudinal studies. In order to further understand the damage of the optic path in MS patients, it is important to document parameters at different points after the inflammatory episode. This may help analyze disease patterns and establish better follow-up intervals [82].

The need for new, fast biomarkers that could offer new diagnostic information has recently been highlighted by the SARS-CoV-2 pandemic. Numerous cases of MS with acute ON onset have been reported to be triggered at the same time as SARS-CoV-2 infection, or in some cases after the vaccine [83,84,85,86]. The pathogenic process may have started before contact with the SARS-CoV-2 virus, or the vaccine, but these elements acted as a precipitating factor that triggered the inflammatory response. OCT-A screening in the normal, asymptomatic population could help identify such sensitive patient profiles.

MS is a demyelinating disease of the CNS that has an increased incidence among young people in developing countries. The OCT structural parameters obtained are measured and compared to normative databases. In spite of the non-invasive character of the device that could support screening in the pediatric population, there are currently no databases incorporated in OCT reports that would be able to highlight any suspicion of increased or decreased layer thickness [87]. Therefore, younger patients are susceptible to errors of interpretation. For this category of patients who have become exposed to a large amount of new risk factors, new implementable databases should be the major target for future research.

## Figures and Tables

**Table 2:** Published studies that analyze OCT-A differences between MS, NMOSD and healthy patients.

Author and Year	Participants and Method	Analyzed Structures	Sub Groups	Study Limitations	Key Findings
Lee et al. [56] 2021	36 MS eyes vs. with 47 NMOSD eyes vs. 36 healthy eyes	RNFL GCL Macular VD ONH VD	No subgroups	Not longitudinal	NMOSD eyes had the lowest structural OCT parameters. IED were found in patients with ON history; Correlation between visual function and ONH VD. Correlation between structural parameters and OCT-A values in MS and between RNFL and OCT-A values in NMOSD.
Aly et al. [57] 2021	41 MS eyes vs. 30 NMOSD eyes vs. 42 healthy eyes	RNFL GCIPL Fovea thickness FAZ area SVP VD DVP VD	MSON vs. MSNON; NMO ON vs. NMO NON	Not longitudinal Not correlated with visual function	ON eyes had a decreased VD and a larger FAZ. No significant differences between VD in MS and NMOSD. NON NMOSD eyes had an increased FAZ while MS and healthy eyes presented normal values. Enlarged FAZ area had a higher correlation with EDSS in NMOSD patients.
Rogaczewska et al. [58] 2021	75 MS eyes vs. 20 NMOSD eyes vs. 40 healthy eyes	Radial peripapillary capillary density	MSON vs. MSNON; NMO ON vs. NMO NON	Not longitudinal Only one analyzed parameter	VD was significantly reduced in NMO ON eyes with a predilection to inferior, superior nasal and nasal superior sectors. RPC in temporal, nasal and ST sectors were decreased in MSON compared with MSNON. Comparing ON and NON eyes, lower RPC VD had temporal and nasal affinity in MS, whereas in NMOSD, 7 out of 8 sectors were affected.
Liu et al. [59] 2021	83 MS eyes vs. 91 NMOSD eyes vs. 34 healthy eyes	RNFL GCL Macular VD FAZ	MSON vs. MSNON; NMOSD ON vs. NMOSD NON	Not longitudinal	No significant FAZ parameters differences between the groups. MS NON and NMOSD NON showed no significant difference in structural OCT parameters when compared to healthy group. NMOSD eyes had lower macular VD compared to MS eyes, especially when comparing NMOSD ON to MS ON. Outer inferior and outer nasal quadrants showed the highest capacity to distinguish between MS and NMOSD patients.

DVP: deep vascular plexus; EDSS: expanded disability status scale; FAZ: foveal avascular zone; GCIPL: ganglion cell–inner plexiform layer complex; GCL: ganglion cell layer; IED: inter-eye differences; MS: Multiple Sclerosis; NMOSD: Neuromyelitis Optica Spectrum Disorder; NON: no prior optic neuritis; OCT-A: Optical Coherence Tomography Angiography; ON: prior optic neuritis; ONH: optic nerve head; RNFL: retinal nerve fiber layer; RPC: radial peripapillary capillary; SVP: superficial vascular plexus; VD: vascular density.

## Data Availability

Data sharing is not applicable to this article.
